# Maternal obesity and ovarian failure: is leptin the culprit?

**DOI:** 10.1590/1984-3143-AR2023-0007

**Published:** 2023-02-20

**Authors:** Yashaswi Sharma, António Miguel Galvão

**Affiliations:** 1 Institute of Animal Reproduction and Food Research of PAS, Department of Reproductive Immunology and Pathology, Olsztyn, Poland; 2 Babraham Institute, Epigenetics Programme, Cambridge, United Kingdom UK; 3 Centre for Trophoblast Research, University of Cambridge, Cambridge, United Kingdom UK

**Keywords:** maternal obesity, leptin, oocyte quality, epigenetics, ovarian leptin resistance

## Abstract

At the time of its discovery and characterization in 1994, leptin was mostly considered a metabolic hormone able to regulate body weight and energy homeostasis. However, in recent years, a great deal of literature has revealed leptin’s pleiotropic nature, through its involvement in numerous physiological contexts including the regulation of the female reproductive tract and ovarian function. Obesity has been largely associated with infertility, and leptin signalling is known to be dysregulated in the ovaries of obese females. Hence, the disruption of ovarian leptin signalling was shown to contribute to the pathophysiology of ovarian failure in obese females, affecting transcriptional programmes in the gamete and somatic cells. This review attempts to uncover the underlying mechanisms contributing to female infertility associated with obesity, as well as to shed light on the role of leptin in the metabolic dysregulation within the follicle, the effects on the oocyte epigenome, and the potential long-term consequence to embryo programming.

## Introduction

Obesity is a complex and progressive disease, well known for its ability to result in a wide spectrum of debilitating co-morbidities, including metabolic disease, type 2 diabetes, cardiovascular disease (Hruby and Hu, 2015), various types of endometrial, breast, or colon cancer (Dağ and Dilbaz, 2015), and reproductive disorders (Kyrou et al., 2018). Infertility is recurrently observed in obese women of reproductive age, who usually present menstrual disorders and anovulatory cycles, lower implantation and pregnancy rates, as well as failed assisted reproductive interventions (Dağ and Dilbaz, 2015). Infertility is, therefore, a prominent co-morbidity of obesity, the aetiology of which remains largely understudied. The impairment of reproductive function in obese females occurs at both central and peripheric levels and can affect either the ovaries or the endometrium (Bellver et al., 2007). Literature comprehensively characterises the major readouts associated with ovarian pathology and failure in obese women, which comprise an excessive accumulation of lipids or lipotoxicity in various ovarian components (Wu et al., 2010), increased endoplasmic reticulum stress and apoptosis (Yang et al., 2012), increased inflammation (Robker et al., 2011), altered mitochondrial function and oxidative stress (Igosheva et al., 2010). Thus, the aforementioned features invariably lead to impaired ovulation and reduced oocyte developmental competence (Robker et al., 2009). Overall, obesity poses a clear threat to ovarian function and the quality of the growing gamete; nonetheless, we lack mechanistic insights and understanding of the molecular mechanisms underpinning such detrimental effects at various cellular levels.

The deleterious effects of obesity result from a major endocrine imbalance that follows the expansion of fat stores. Amongst several hormones being dysregulated in the course of obesity, leptin, a key bioactive peptide largely secreted from the white adipose tissue (adipokine) (Friedman and Halaas, 1998), has a strong association with both obesity and reproduction (Tong and Xu, 2012). In fact, mice with a homozygous mutation for the leptin-producing gene *ob* (obese gene), were shown to develop both obesity and infertility (Zhang et al., 1994). Leptin concentrations rise rapidly in circulation in obese specimens, since leptin circulating levels are positively correlated with body fat mass (Considine et al., 1996; Maffei et al., 1995). Initially, leptin was shown to act as an important neuroendocrine regulator of food intake and energy homeostasis (Farooqi and O'Rahilly, 2009; Zhang et al., 2005). Nonetheless, like other adipokines, its pleiotropic actions were soon reflected at various levels, such as the regulation of the immune system, haematopoiesis, angiogenesis (Matarese et al., 2006), cognition and bone metabolism (Dalamaga et al., 2013) and reproduction (Childs et al., 2021). In recent years, leptin has engendered a great deal of interest in its regulatory role in reproductive tract and fertility (Castracane and Henson, 2003). In women, leptin is known to be associated with all stages of reproductive age - puberty, menstrual cycle, pregnancy, as well as menopause. A number of informative and well-conceived reviews have elaborated on the particular roles of leptin at each one of the above-mentioned stages (Brannian and Hansen, 2002; Pérez-Pérez et al., 2015; Wołodko et al., 2021), reiterating the importance of leptin physiological actions in the control of female fertility. Importantly, under physiological conditions, leptin signals within a narrow concentration range, and excessive or insufficient levels of leptin may compromise fertility and ovarian function (Childs et al., 2021). For instance, conditions of hypoleptinemia, such as in hypothalamic amenorrhoea (Miller et al., 1998), have been characterised by anovulation and dysregulation of the oestrous cycle (Chou and Mantzoros, 2014). Interestingly, fertility was shown to be restored in such patients after leptin treatment(Welt et al., 2004). Conversely, hyperleptinemia observed during obesity was also associated with polycystic ovarian syndrome, hypogonadism associated with type 2 diabetes and infertility, (Chou and Mantzoros, 2014) by mechanisms yet to be fully understood. Of particular importance, obese women are known to present high levels of circulating leptin, the state of hyperleptinemia, and often developing insensitivity to exogenous administration of leptin, a state known as leptin resistance. Therefore, hyperleptinemia and leptin resistance are two major features of obesity likely to drive the detrimental effects of energy surplus on ovarian function. This dichotomy in leptin signalling throughout obesity dramatically affects ovarian function. In early obesity, the establishment of a rapid hyperleptinemia and increased leptin signalling may affect ovarian function particularly through the negative impact on folliculogenesis, altered steroid synthesis and secretion in the growing follicle, and oocyte maturation (Brannian and Hansen, 2002). Conversely, the establishment of ovarian leptin resistance observed in late obesity, may result in perturbations in ovulation (Pérez-Pérez et al., 2015) or increased primordial follicle recruitment, leading to reduced reproductive performance and premature ovarian failure (Moslehi et al., 2018). Such a complex set of actions, alongside the inherent intricacies of ovarian function regulation, portrays leptin as a key but challenging signalling system to study during obesity. Consequently, little is known about the impact of altered levels of leptin signalling in the ovary and the gamete. In particular, it is still unclear whether local changes in leptin signalling can directly impact the oocyte epigenome, posing therefore the risk of transmission of such epimutations to the embryo, and potentially jeopardising early embryo development and reprogramming events.

**Figure 1:**
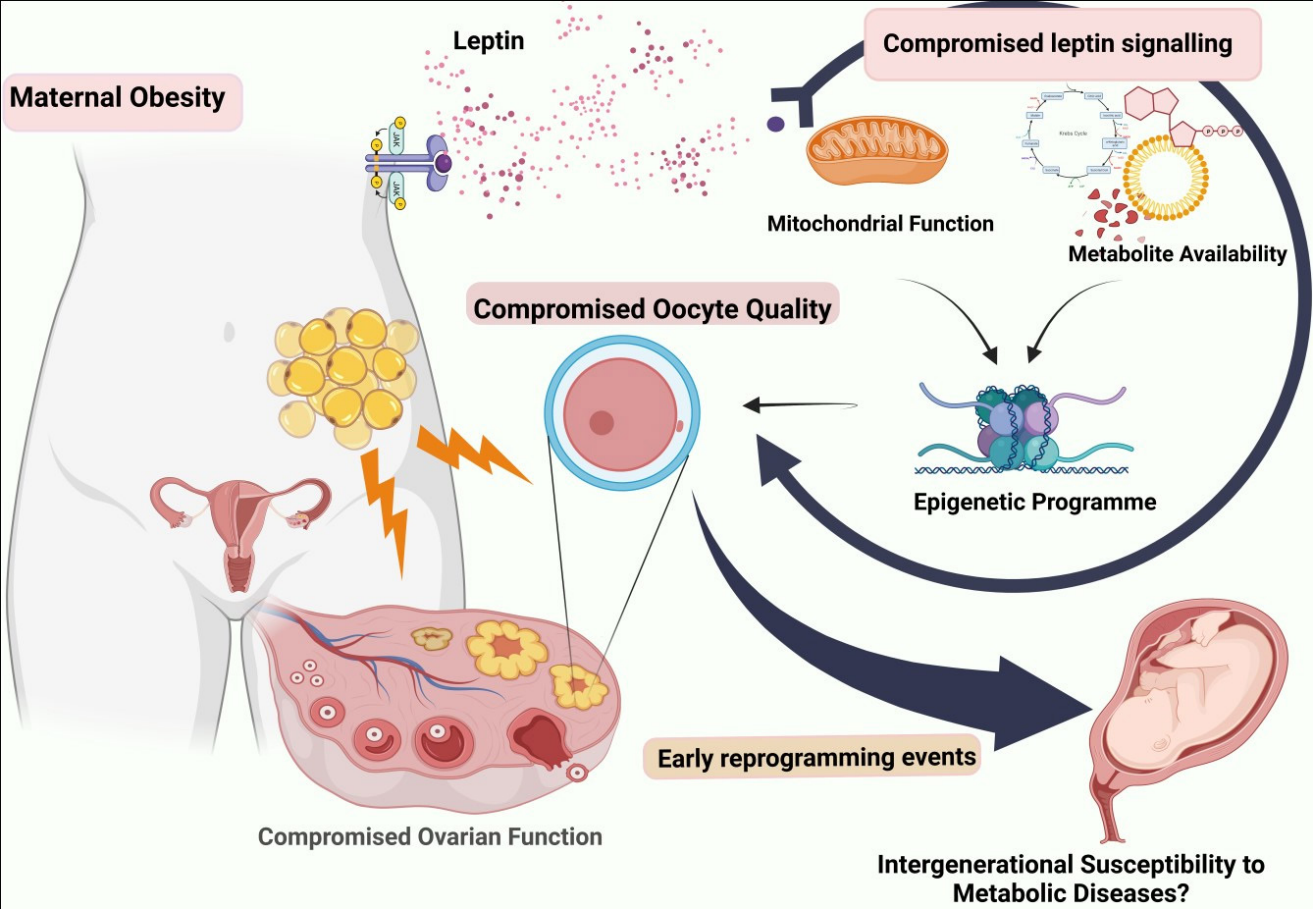
Maternal obesity and the putative impact of altered ovarian leptin signalling on oocyte growth and long-term effects for embryo development and offspring health. With the increase in fat stores in the body, leptin is produced in large amounts leading to hyperleptinemia and subsequent leptin resistance in the ovary. Local perturbations in leptin signalling may lead to changes in oocyte metabolic profile and mitochondrial function, which may critically affect oocyte quality (Ge et al., 2012), affecting the oocyte metabolic and epigenetic legacy which, in turn, may affect early reprogramming events in the embryo and offspring susceptibility to disease in adulthood. *Created with BioRender.com*.

Collectively, growth and maturation of the female gamete are highly demanding processes, requiring an optimal interplay between maternal nutritional state and other environmental factors, which will invariable control nuclear, cytoplasmic and epigenetic maturation (He et al., 2021), ultimately ensuring the adequate transfer of genetic and epigenetic information required for embryonic development. Since, the environment in which the oocyte grows and develops critically determines its quality, it is extremely relevant to understand how maternal metabolic performance may affect such environment, both under physiological and pathological conditions. One such promising factors controlling metabolite availability and energetic performance is leptin. The present review, revisits the metabolic roles of leptin in various cellular contexts, which may pose deleterious consequences to oocyte and early embryo development in the context of maternal obesity (Figure 1). It sheds light upon the missing links in literature to better understand the crosstalk between obesity, altered ovarian leptin signalling, and putative consequences for oocyte development, particularly instigating the effects on oocyte metabolome and epigenome and further outcomes for early embryo development.

## Obesity, leptin signaling, and the establishment of leptin resistance in the ovaries

Leptin modulates female reproductive tract through endocrine and neuroendocrine mechanisms, but may also locally regulate ovarian activity, particularly controlling steroidogenesis (Hausman et al., 2012), folliculogenesis (Brannian and Hansen, 2002), and luteal function (Galvão et al., 2012). Under physiological conditions, leptin signals through a single-spanning transmembrane protein receptor belonging to the class I cytokine receptor superfamily (Tartaglia et al., 1995). Although at least five isoforms of the receptor have been identified, the canonical pathway involves the activation of Janus kinase (JAK)/signal transducer and activator of transcription (STAT) signalling through the long isoform of the receptor, called leptin receptor b (ObRb) (Banks et al., 2000). The non-canonical signalling pathway includes the activation of insulin receptor substrate (IRS)/phosphatidylinositol 3 kinase (PI3K)/protein kinase B (Akt) and mitogen-activated protein kinase (MAPK)/extracellular signal-regulated kinase (ERK) signalling pathways (Bjørbæk et al., 1997, 2001; Hegyi et al., 2004). With the expansion of fat stores in the course of obesity, the levels of leptin in circulaiton dramatically rise and result in the dysregulation of the aforementioned signalling pathways (Myers et al., 2010). Although ideally it might be expected that leptin acts as a true ‘anti-obesity’ hormone promoting satiety, reducing food intake and increasing energy expenditure (Myers et al., 2010), its ability to modulate adiposity can be forestalled by perturbations in the activation of the signalling pathway, often characterised by hyperleptinemia. The condition termed ‘leptin resistance’ may be mediated by: i) the down-regulation of leptin receptors, ii) the presence of receptor defects, iii) deficiencies in the secretion or circulation of the protein that compromises its bioavailability, or iv) its inability to reach the target tissue, for instance, crossing the blood-brain barrier (Castracane and Henson, 2003). The most common reason for leptin resistance, however, is the failure of the intracellular signalling cascade of the ObRb receptor (Enriori et al., 2007), widely mediated by molecules such as the suppressor-of-cytokine-signaling-3 (SOCS-3) protein (Bjørbaek and Kahn, 2004; Myers, 2004; Rabe et al., 2008) and the phosphotyrosine phosphatase-1B (PTP1B) (Bence et al., 2006; M. G. Myers et al., 2010). The ensuing resistance is specifically termed ‘cellular leptin resistance’. With the hyperactivation of the leptin signalling and the resulting increase in phospho-signal transducer and activator of transcription 3 (pSTAT3), the gene expression of SOCS-3 is upregulated, which in turn blocks tyrosine and Janus kinase-2 (JAK2) phosphorylation in a classic feedback inhibition pathway (Bjørbæk et al., 2000). Similarly, the upregulated expression of PTP1B due to STAT3 signalling, dephosphorylates JAK2 to block leptin signalling (Cheng et al., 2002; M. P. Myers et al., 2001). More recently a number of novel molecules have been described to mediate leptin resistance centrally, such as protein tyrosine phosphatases (PTPs), brain-derived neurotrophic factor (BDNF), myeloid differentiation factor 88 (MyD88), methyl-CpG-binding protein 2 (MeCP2), I-kappa-B kinase epsilon (IKKε), extracellular signal-regulated kinases (ERKs), mitofusin 2 (MFN2), histone deacetylase 5 (HDAC5), withaferin A, c-Jun N-terminal kinases (JNKs), activating transcription factor 4 (ATF4) (J. Liu et al., 2018), each with its own proposed mechanism of action. Ultimately, increased circulating levels of leptin during obesity will culminate with the dysruption of the signalling pathway in an organ specific manner.

The state of leptin resistance has unique features, with regard to ovarian function. As shown by studies in mice treated with high fat diet (HFD), leptin resistance sets in phases, in which initially the sensitivity to peripheral leptin injection is maintained, followed by peripheral insensitivity but adequate response to central leptin injections and finally a late stage in which the mice develop central leptin resistance (Enriori et al., 2006). Recently it was also shown that leptin resistance may be organ specific. For instance, we have shown that the establishment of ovarian leptin resistance in mice after 16 weeks of diet induced obesity (DIO), followed an increase in leptin signalling in the ovaries of mice under DIO for 4 weeks (Wołodko et al., 2020). Such organ specific response has also been shown in the hypothalamus (Münzberg et al., 2004; Ozcan et al., 2009) and the liver of both mice and humans (Brabant et al., 2005). Nonetheless, other organs like the heart and kidney are known to maintain its responsiveness to leptin throughout obesity (Mark et al., 2002; Morgan et al., 2008). Thus, the regulation of leptin signalling in the course of obesity seems to be organ dependent, and particularly for the ovaries associated with the levels of obesity and pregression of the disease. Overall, leptin resistance is either an adaptive response or a pathological state (Tups, 2009), which marks the onset of impaired leptin signalling under conditions of leptin excess, such as during obesity.

## Balanced leptin signalling: a pre-requisite for oocyte metabolic homeostasis

### Oocyte maturation

The successful fertilisation of the female gamete and further embryo development requires the accomplishment of major steps during oogenesis, the nuclear, cytoplasmic, and epigenetic maturation. (Eppig et al., 2004). The process involves events like meiotic resumption and metaphase II arrest, accumulation of mRNA, proteins, and nutrients which will enable the genomic modifications ensuring correct gene expression programs during embryo development (Eppig et al., 2004; Watson, 2007). Oocyte growth and maturation are, therefore, highly orchestrated events that require an optimal interplay between intrinsic signals and nutritional and environmental factors (Hunt and Hassold, 2008). Among the various factors controlling the development, competence and quality of the female gamete, oocyte metabolism is widely known to play key roles (Sirard, 2011), providing the energy required for meiotic progression, buffering between intracellular redox and osmotic potential and, most importantly, providing the building blocks for growth (Watson, 2007). Thus, oocyte growth requires an active synthesis of metabolites and metabolic enzymes for the regulation of multiple cellular events. The demand for such metabolites and energy substrates is cratered by both the oocyte machinery and the surrounding cumulus cells (CCs) through specialized membrane connections called gap junctions (Russell et al., 2016; Sugiura and Eppig, 2005). Specific processes in the oocyte require a characteristic metabolic milieu for a successful developmental progression. For instance, the maturation of the oocyte was associated with a state of diminished bile acid biosynthesis, decreased levels of polyunsaturated fatty acids (PUFA), but increased availability of nucleotides and one-carbon metabolism (Li et al., 2020). Such metabolic control is mostly dependent upon the activity of intracellular substrates and enzymes present in the oocytes, other intracellular mediators, the transport across the plasma membrane, and nutrient availability from the follicular environment (Kurus et al., 2013). Overall, metabolite availability dictates how efficiently events like oocyte growth, meiosis, or epigenetic programming are coordinated in a developmentally competent oocyte.

### Leptin metabolic roles

Leptin has always been closely related to metabolism, with several lines of evidence indicating its regulatory role over carbohydrates, lipids, and protein metabolism (Pereira et al., 2021). Literature has shown that leptin controls glucose homeostasis at different levels, stimulating glucose uptake in the skeletal muscle, heart, and brown adipose tissue (Minokoshi et al., 2012), potently suppressing circulating insulin levels, while increasing gluconeogenesis, decreasing glucagon and attenuating glycogen synthesis (D’souza et al., 2017). Furthermore, leptin has also been shown to regulate lipids and protein metabolism. A recent study on patients with congenital deficiency of leptin showed that treatment with leptin promoted major metabolic changes, such as lipid catabolism involving fatty acid oxidation and cholesterol breakdown (Lawler et al., 2020). The regulatory role of leptin in fatty acid oxidation was also supported by the findings of Kircherlber and colleagues, who reported a negative association between acylcarnitines and acetylcarnitine levels with plasma leptin concentrations (Kirchberg et al., 2017). Another study in the skeletal muscle by Minokoshi and colleagues supported leptin’s direct influence on fatty acid oxidation by reversal of the inhibitory action of carnitine palmitoyltransferase I (CPT-1) (Minokoshi et al., 2012). Similarly, leptin concentrations in the plasma were positively associated with the presence of a number of amino acids, like alanine and asparagine (Kirchberg et al., 2017). Hence, leptin seems control the metabolism of macronutrients. It is, therefore, highly plausible that changes in circulating levels of leptin may promote important changes in the availability of metabolites, their precursors and catabolic products.

### Leptin metabolic roles and the ooocyte

The ovaries and the oocyte are no exception with regard to leptin regulatory actions on energy homeostasis. Both the ovarian cells (Lin et al., 2000; Ruiz-Cortes et al., 2000) and the oocytes (Ryan et al., 2002; Cervero et al., 2004) of several animal species and humans are known to express the different forms of leptin receptors including the ObRb mRNA and protein. This suggests the ability of ovarian cells to respond directly to fluctuations in circulating levels of leptin. Therefore, under conditions of altered leptin signalling such as in obesity, the metabolism in ovarian somatic cells, as well as the gamete, are susceptible to dysregulation.

#### Glucose metabolism

Glucose is essential for oocyte development, and is metabolised in a concerted way between the oocyte and granulosa cells (GCs) by glycolysis, the pentose phosphate pathway (PPP), hexosamine biosynthesis pathway (HBP) or the polyol pathways (Sutton-McDowall et al., 2010). Pyruvate is the preferred energy substrate for the oocytes, but given the low glycolytic rate and capacity for glucose uptake and transport (Harris et al., 2007; Saito et al., 1994), oocytes are dependent upon GCs to access such metabolic intermediates. Pyruvate is also known to be pivotal for oocyte maturation (Johnson et al., 2007) and successful meiotic division. In fact, it was previously corroborated that enhanced tricarboxylic acid (TCA) cycle activity and pyruvate oxidation during this stage favoured the accomplishment of meiosis (Li et al., 2020). Glucose metabolites were also shown to be essential for nucleic acid and purine synthesis, maintenance of redox state, CCs expansion, cell signalling, and the regulation of oocyte nuclear maturation (Sutton-McDowall et al., 2010). The oocyte is however highly sensitive to changes in the availability of glucose, with either high or low levels of glucose resulting in precoucious resumption of nuclear and cytoplasmic maturation, failed fertilization and impaired embryo development (Sutton-McDowall et al., 2010). On one hand, low glucose levels may lead to reduced *de novo* purine synthesis, depleted hyaluronic acid and low energy availability in the oocytes, while on the other hand, high glucose levels may lead to increased reactive oxygen species (ROS) and untimely maturation of the oocyte (Sutton-McDowall et al., 2010). Moreover, the intrafollicular hyperglycemia that ensues under conditions like diabetes, obesity, and poor diet was shown to be detrimental to oocyte viability in mice (Moley et al., 1998). Specifically concerning obesity, higher body mass index (BMI) was associated with alterations in the follicular fluid composition showing increased follicular insulin, glucose and lactate concentrations (Robker et al., 2009). Furthermore, leptin levels were also shown to increased in the follicular fluid of obese women, suggesting that ovarian follicular environment mirrors the systemic alterations seen during obesity (Mantzoros et al., 2000). Indeed, it has been suggested that with the simultaneous increase in the circulating levels of leptin and glucose, the glucose metabolism is altered in various tissues including ovary and possibly the the oocyte (Silva et al., 2012). Such changes in the metabolism, particularly that of glucose, have also been linked to establishing epigenetic memory, with reports showing glucose-induced alterations of posttranslational modifications to histones, including methylation and acetylation (Pirola et al., 2010). Furthermore, it was also reported the strong crosstalk between leptin and insulin signaling pathways during the regulation of glucose metabolism (Koch et al., 2010). Leptin increase has been shown mitigate the deleterious effects of high glucose on oocyte development, mostly through the downregulation of insulin-like growth factor 1 (IGF1) expression in CCs, and the upregulation of insulin receptor substrate 1 (IRS1) in oocytes (Silva et al., 2012). High leptin was also shown to alter glucose metabolism in cumulus-oocyte-complexes (COCs) by downregulation of glucose transporter 1 (GLUT1) (Silva et al., 2012). Finally, leptin was also shown to improve glycolytic activity in the oocytes through IRS1 upregulation and IGF1 receptor action leading to phosphatidylinositol 3-kinase activation and thereby significantly influencing glucose metabolism in different tissues including the ovary(Poretsky et al., 1999). Collectively, these findings indicate that metabolism of glucose and leptin action are closely interconected in the oocytes. Glucose and its metabolites are indispensable for normal oocyte growth and quality, and therefore any alteration in its metabolism, particularly associated with fluctuations in leptin signalling in obese individuals, may cause decreased competence and subfertility.

#### Lipid metabolism

Fatty acids, stored in the form of triglycerides, are the major energy reserves in the oocytes. The intracellular lipid levels change throughout oocyte growth and development, especially during maturation, in a species-specific manner (Gu et al., 2015). As reported by a recent study characterising the metabolome of mouse oocytes, a 3- to 4-fold increase in carnitine and palmitoyl-carnitine content was reported in oocytes around the time of meiotic resumption (Li et al., 2020), while lipid-metabolism related products including cholesterol and arachidonic acid were shown to be depleted as the oocytes progress through meiosis(Li et al., 2020). Mitochondrial oxidation follows the breakdown of fatty acids by lipases in the oocyte or CCs and releases acetyl-CoA, which can then enter the TCA cycle to produce energy in the form of adenosine triphosphate (ATP) (Dunning et al., 2014). The importance of oocyte lipid metabolism, especially that of beta-oxidation can be ascertained by a number of studies reporting the reduction of embryo viability (Ferguson and Leese, 2006) and inhibition of meiotic oocyte resumption (Downs et al., 2009) following inhibited beta-oxidation activity. Conversely, enhanced oocyte nuclear and cytoplasmic maturation was confirmed under conditions of active lipid metabolism (Dunning et al., 2011). Furthermore, fatty acid metabolites were also shown to be involved in cell signalling events, regulating oxidative stress, membrane composition, and controlling gene expression in the female gamete (McKeegan and Sturmey, 2011). For instance, diacylglycerol (DAG), which acts as a secondary messenger in the triphosphoinositol (IP_3_) /DAG pathway, is known to facilitate the activation of protein kinase C (PKC), which has been implied to play specific roles in oocyte development, such as meiotic resumption, spindle organization, and activation (Kalive et al., 2010). Other lipid metabolites such as ceramide, are known to promote meiosis ressumption (Strum et al., 1995), while fatty acids binding to nuclear receptors and transcription factors (Sampath and Ntambi, 2005), have been linked to successful embryo development and female fertility(al Darwich et al., 2010; Cui et al., 2002). Given the crucial roles that lipids play in all stages of normal oocyte development, the importance of an optimal state of lipid metabolism as well as that of its regulator is insurmountable.

Leptin is a well established regulator of lipid metabolism, known to stimulate lipolysis and fatty acid oxidation in many cell types (Reidy and Weber, 2000), including the components of the ovarian follicles (Zhang et al., 2015). Leptin is also known to be a key modulator of cellular triacylglycerol content (Reidy and Weber, 2000), which, as previously discussed, is the main energy source described for the oocytes. Moreover, it has also been reported that leptin is an important factor linking fatty acid β-oxidation with oocyte maturation, through the JAK-STAT pathway (Zhang et al., 2015). However, under conditions of obesity, ovaries and oocytes are known to accumulate excessive lipids resulting in increased oxidative stress and ovarian inflammation (Cardozo et al., 2016; Gu et al., 2015). This can be a result of the putative pathogenic role of hyperleptinemia in lipid accumulation (Shen et al., 2019). Although different mechanisms may be at play, the downregulation of master regulators of lipid metabolism like Sterol regulatory element binding protein 1 (SREBP-1c) under high leptin levels has been previously reported(Shen et al., 2019). Such long-term exposure to altered lipid levels may therefore impair the oocyte function or their somatic counterparts (T. Liu et al., 2022). Finally, as shown by studies on polycystic ovarian syndrome (PCOS) (Khan et al., 2021), associated most commonly with obesity, impaired lipogenesis and lipolysis events are commonly seen in the ovaries. This could be also ascribed to leptin resistance during obesity which impairs leptin’s peripheral role in regulating lipid metabolism in cells (Sáinz et al., 2015). Generally, leptin is well known to control lipid oxidation and regulate triglyceride cellular homeostasis, critical for energy generation and the development of the oocyte. Therefore, changes in leptin levels during obesity levels can potentially affect oocyte fatty acid oxidation and energy provision during critical phases of oocyte growth and developmental competence.

#### Amino-acid metabolism

Oocytes access amino-acids via their unique transport systems like β, L, GLY, x_c_^−^, and b^0,+^ (Pelland et al., 2009). Alternativelly, they also depend on GCs and CCs for the uptake of specific amino acids like L-alanine, glycine, taurine, and lysine. Amino acids are utilised in the oocyte as substrates for energy, for protein synthesis, to facilitate osmosis, and also as redox buffering elements (van Winkle, 2001). Specifically, they are also known to support the development of preimplantation embryos, while in post-implantation embryos, amino acids are known for their roles in fostering viable embryos and supporting early embryo cleavage (van Winkle, 2001). For instance, the addition of glutamine to the oocyte culture medium turned out as an efficient energy substrate, improving oocyte maturation (Songsasen and Wildt, 2007) and initiating meiotic resumption (Downs and Hudson, 2000). Glutamine and other amino acids like aspartate and valine have also been shown to avert polyspermy in pigs (Hong and Lee, 2007) and glycine has been implicated in exerting unique cell volume regulatory mechanisms (Baltz and Tartia, 2010). Typical temporal metabolite profiles of amino acids are evident during the course of oocyte growth and development, with different amino acid subsets increasing in abundance at particular stages of development, while declining during others. For example, a significant increase in the levels of serine, glutamate and histidine was evidenced during meiotic resumption, whereas their availability was shown to be decreased post-maturation (Li et al., 2020). Amino acids may act as key components in the synthesis of *de novo* purine and pyrimidines, guanosine triphosphate (GTP), nicotinamide adenine dinucleotide (NAD^+^) and are the sources of carbon and fixed nitrogen (Sturmey et al., 2008). Besides their metabolic regulation and role in protein synthesis, some amino acids were shown to be involved in the regulation of DNA methylation. As an exmple, methionine along with folate and vitamin B12 were shown to be important co-factors that integrate the methylation cycle (Gilbody et al., 2007). Additionally, the Serine-Glycine-One-Carbon (SGOC) pathway, which involves the folate and the methionine cycles, was shown to be upregulated during meiotic resumption of the oocytes (Li et al., 2020), with SGOC fuelling methyltransferase activity (Reina-Campos et al., 2020) and shaping the epigenetic landscape of the oocytes. Hence, protein metabolism is one of the most diverse yet important biochemical process that dictate the developmental potential of the oocyte. Amino acids and their metabolites are equally susceptible to alteration in their normal metabolism under conditions of obesity (Short et al., 2019; Zhou et al., 2013), most likely driven by conditions of hyperleptinemia. As shown by a metabolomic study on lipodystrophy patients receiving leptin treatment, a drastic change in protein and amino acid catabolism was evident leading to overall increased protein turnover (Grewal et al., 2020). The study reported that leptin caused an increase in markers of protein degradation, like gamma-glutamyl amino acids, 3-methylhistidine, and N-acetyl-3-methylhistidine along with metabolites involved in urea cycle (Grewal et al., 2020). This can be particularly detrimental in the context of ovarian funciton and oogenesis, since increased protein catabolism and the resulting presence of high concentrations of ammonium and urea have been shown to reduce embryonic development and promote sustained metabolic stress in the surviving embryos (Sinclair et al., 2000). Also, the altered profiles of plasma amino-acids observed during obesity, can further dysregulate carbohydrate metabolism in oocytes. For instance, high levels of leucine and tyrosine in the plasma are known to increase the levels of glucagon (Rooke et al., 2009) and also stimulate insulin release (Calbet and MacLean, 2002). This is particularly relevant for the environment of the growing gamete, as the free amino acid profile in plasma is broadly similar to follicular fluid free amino acid composition (Orsi et al., 2005). Furthermore, the total free amino acid concentration is proven to be very closely related to oocyte quality (Rooke et al., 2009). In conclusion, increased levels of leptin, or leptin resistance, may be at least partly responsible for the oocyte abnormalities taking place under conditions of metabolic dysregulation during obesity.

Overall, in this section we have seen that obesity leads to a state of systemic and local metabolic dysregulation, which is likely to be affected by altered levels of circulating leptin. Through its metabolic actions on various substrates, leptin may alter the levels as well as profiles of available metabolites. In the ovarian context, such events may impede the physiological development of the gamete, through direct actions on oocytes or through the metabolic dysregulation of the surrounding GCs and CCs. In fact, our recent study on the transcriptome of CCs isolated from DIO mice revealed dramatic changes in the expression of genes particularly involved in cellular trafficking and cytoskeleton organisation of the CCs, which were also found in the CCs of mice treated with leptin (Wołodko et al., 2020). Such changes can render the CCs inefficient in supplying the oocytes with the metabolites or metabolic precursors it demands, compromising the oocyte developmental competence. For instance, leptin treatment of COCs during in vitro maturation at 100 ng/ml of concentration, was reported to downregulate the expression of GLUT1 in CCs (Silva et al., 2012). In fact, the oocyte relies on the CCs critical supply of glucose and its metabolites, cholesterol biosynthesis as well as for some amino-acids (Su et al., 2009.). Moreover, the potential of leptin to influence the function of CCs have been also studied in bovine oocytes, where an optimal level of leptin was shown to enhance developmental potential of oocytes via CC-dependent mechanisms (Paula-Lopes et al., 2007). Thus, owing to its systemic and ovarian roles in metabolic homeostasis, fluctuations in leptin circulating levels observed during maternal obesity may affect the oocyte metabolism, quality and fertility outcomes. In a nutshell, the periconceptional period encompassing the active stages of oocyte growth and development is sensitive to metabolite availability in the germ cell. The presence of adequate metabolic substrates at physiological levels ensures that nutritional needs are met, as well as critical events for the quality and competency of the female gamete are maintained. Any form of deficiency or excess of macro- or micro-nutrients or other metabolic substrates during this period can therefore lead to reduced fertility, altered foetal development and compromised long-term offspring health.

## Leptin and the oocyte mitochondrial function

Highly acclaimed as the ‘powerhouse of the cell’, mitochondria are indispensable for oocyte growth and development, being a critical indicator of oocyte quality (Schatten et al., 2014). Therefore, we dedicate a chapter specifically to the putative effects of leptin signalling dysregulation on mitochondrial function in oocytes during obesity.

The developing oocyte and the surrounding follicular cells are highly reliant on the biosynthetic precursors and the energy produced by mitochondrial oxidative phosphorylation (Qi et al., 2019). Hence, ATP, the main product from oxidative phosphorylation, is particularly important for active transcription and translation, which drives oocyte maturation (Kirillova et al., 2021). Furthermore, optimal mitochondrial function is required for the formation of the meiotic spindles before and during oocyte activation (Benkhalifa et al., 2014), as well as for the maintenance of redox homeostasis (Spinelli and Haigis, 2018). Given the pivotal roles played by the mitochondria, it is critical that oocytes contain a minimum threshold number of mitochondria, and adequate copies of mtDNA (Wai et al., 2010). In other cellular contexts, mitochondria play a central role in the regulation of cell senescence and death, facilitating cell signalling and the biosynthesis of compounds like nucleotides, fatty acids, cholesterol, amino acids, and heme (Spinelli and Haigis, 2018). Importantly, in immature oocytes, mitochondria are initially transcriptionally and bioenergetically silent (Allen and Paula, 2013), which certainly minimises the occurrence of mitochondrial DNA (mtDNA) mutations (Allen and Paula, 2013). Conversely, after fertilization, mitochondrial activation is necessary to protect the oocyte from oxidative damage and support early embryo development (Qi et al., 2019). In fact, mitochondrial inheritance is exclusively maternal and oocyte-derived mitochondria give rise to the entire mitochondrial content present in the various tissues of the offspring (McPherson et al., 2015). As a result, coordinated regulation of mitochondrial activity in oocytes ensures not only the competence of the gamete but also the successful development of the embryo and metabolic performance in the offspring.

Numerous reports have recently described the dramatic impact of maternal obesity on oocyte mitochondrial activity. Studies in mice have shown that diet-induced obesity alters oocyte mitochondrial morphology, such as decreased number of cristae and vacuoles (Luzzo et al., 2012), increased mtDNA copy number and higher mitochondrial biogenesis (Igosheva et al., 2010; Luzzo et al., 2012), or changes in mitochondrial potential (Igosheva et al., 2010; Wu et al., 2010). It was also observed that energetic surplus in mothers is associated with increased ROS formation, and altered spatial distribution of mitochondria in the oocyte (Igosheva et al., 2010). Hence, spindle and chromosome alignment defects leading to aneuploidy, failed oocyte maturation, poor fertilization rates and abnormal embryo development frequently reported in obese women may be explained by the underlying mitochondrial dysfunction in the oocytes from obese mothers.

Importantly, mitochondrial metabolites were shown to affect gene expression regulation and promote epigenetic changes during oocyte maturation and embryo development (Ge et al., 2015; Matilainen et al., 2017; Whidden et al., 2016). For instance, mitochondrial metabolites of the TCA cycle such as ATP, alpha ketoglutarate (α-KG), and citrate were shown to alter chromatin configuration which was associated with gene expression (Qi et al., 2019). Additionally, citrate is known to facilitate histone acetylation and drive gene expression by changes in chromatin conformation. This was shown to be facilitated by the conversion of citrate into acetyl-CoA, with the donation of the acetyl group to histone acetyltransferases (HATs) (Montgomery et al., 2015). Similarly, α-KG is known to promote DNA demethylation, working as a co-factor of ten-eleven translocation (TET) enzymes, which in turn are known to catalyse the hydroxylation of methylated cytosines in the genome (Qi et al., 2019). With regard to histone methylation, α-KG was shown to act as a crucial co-factor of histone demethylases (HDMs) (Kooistra and Helin, 2012). Another metabolite, the S-adenosyl methionine (SAM), a universal methyl- donor and a common substrate for numerous enzymatic reactions, is known to be originated from the folate cycle and ATP generated by the mitochondria (Qi et al., 2019). In fact, SAM is a critical regulator of DNA methylation as is often utilised as a coenzyme involved in the transfer of methyl groups (Smith and Denu, 2009). As previously reported, the availability of mitochondrial substrates like SAM are known to maintain human embryonic stem cells (hESCs) pluripotency, as decreased levels of SAM in culture resulted in cell differentiation (Sperber et al., 2015). Also, the energy required for the modulation of changes in chromatin configuration and specific binding of the chromatin remodelling complexes is largely ATP-dependent (Flaus and Owen-Hughes, 2011). Finally, acetyl-CoA dependent HAT activity was also shown to control oocyte maturation and the activation of follicular reserve (Yin et al., 2017). This suggests that mitochondria, and the metabolites generated through their activity, not only play major physiological roles in regulating energy homeostasis but also maintain the stability of both genetic and epigenetic signatures in gametes and developing embryos.

Of great relevance within the scope of obesity, but largely understudied, is the potential impact of altered local leptin signalling on mitochondrial function in the oocyte from obese mothers. This seems plausible, especially considering that leptin treatment was shown to increase mitochondrial metabolism and ATP production, decrease oxidative stress, promote mtDNA replication, and increase mitophagy, generally affecting mitochondrial function in oocytes (Blanquer-Rossellõ et al., 2015). In addition, it has also been suggested that leptin can influence the routes of mitochondrial ATP production, since the ATP production in db mice lacking functional leptin receptors was less reliant on glycolysis, but rather on beta-oxidation (J. Park et al., 2010). A number of other studies on muscle, endothelial cells, and adipocytes have also revealed the stimulatory role of leptin on fatty acid oxidation, glucose uptake and ROS production (Yamagishi et al., 2001; Minokoshi et al., 2002; Luo et al., 2008), which resulted in increased mitochondrial activity (Henry et al., 2011). Therefore, under the influence of leptin, mitochondrial metabolism appears to be enhanced, with subsequent changes in metabolites and major outcomes for gene expression regulation and epigenetic changes. For instance, the availability of acetyl-CoA is mostly dependent on the rate of mitochondrial metabolism. Under increased metabolism, the actyl-CoA in excess facilitates histone acetylation, which has been associated with active gene transcription (Menzies et al., 2016). Furthermore, glucose derived acetyl-CoA supplies most of the acetyl group for histone H4K16 acetylation (Morrish et al., 2010), as well as to histone acetyltransferases enzymes (Montgomery et al., 2015), the availability of which can be influenced by leptin regulatory effects on mitochondrial activity (Blanquer-Rossellõ et al., 2015; Henry et al., 2011). Thus, the epigenome can be modulated in response to the availability of essential metabolites, which can in turn be regulated by leptin.

Mitochondria are one of the most relevant organelles for oocyte development. Obesity clearly affects the oocyte mitochondrial function, leading to mitophagy and impaired oocyte function and quality. Such changes are accompanied by the dysregulation of oocyte gene expression and the epigenetic program and may be determined by local changes in leptin signalling, which can directly modulate oocyte mitochondrial activity and function. Thus, altered leptin signalling and associated impaired mitochondrial function most certainly affect the oocyte quality and contributes to the pathogenesis of ovarian failure and infertility in maternal obesity.

## Leptin and methylation changes

We have previously discussed the putative role of leptin on epigenetic actions mostly through the modulation of metabolite availability and mitochondrial function. Despite being scarce, evidences of leptin direct actions on epigenetic machinery start getting noticed. Leptin has been recently associated with changes in DNA methylation, and post-transcriptional histone modifications, as well as the regulation of microRNA (miRNA) (Wróblewski et al., 2019) in various cellular contexts. Therefore, we presently discuss the most up-to-date studies on leptin-mediated epigenetic changes.

Studies related to carcinogenesis showed leptin involvement in the modulation of important enzymes controlling epigenetic processes. For instance, in a study on human colon cancer cells, leptin was found to up-regulate the expression of histone deacetylase enzyme sirtuin 1 (SIRT1) (Song et al., 2018) . In another study in ovarian cancer, it was reported that leptin modulated HDACs gene expression, in which class I and II HDACs were increased in OVCAR-3 cells, while class II HDAC expression was increased in folliculoma cells (Fiedor et al., 2018). Importantly, animals with dysregulated leptin signalling, the diabetic mouse (db) and obese mouse (ob), showed lower expression of SIRT1 in colon cells, suggesting its involvement in leptin-induced pathogenesis of colon carcinogenesis (Song et al., 2018). In another study, leptin was shown to induce the progression and chemoresistance of pancreatic adenocarcinoma by affecting the levels of HDACs and the miRNAs miR21 and miR200a/c in tumors, promoting cancer cell survival and division by acting as a proliferative factor (Tchio et al., 2016). Finally, a recent study on rat adrenal cells revealed that leptin demethylated the promoter of a cation channel- Trpm7 (transient receptor potential melastatin 7) and also induced posttranslational modifications of histone proteins (H3K4me3, H3K27ac and H3K27me3), leading to increased Trpm7 transcription via LEPRb-dependent STAT3 activation (Yeung et al., 2021). Thus, leptin was recently shown to directly regulate the activity of enzymes controlling histone post-translational modifications.

Concerning the direct regulation of DNA methylation, a number of studies have evidenced the ability of leptin to control *de novo* DNA methylation. Leptin was shown to induce changes in the methylation of CpG sites in the *Pomc* promoter, the precursor of the melanocyte-stimulating hormone. Indeed, animals treated with leptin during lactation showed hypomethylation of CpG dinucleotides in a specific region of *Pomc* promoter in the hypothalamus, when exposed to high-fat diet conditions later in life (Palou et al., 2011). This suggests the putative role of leptin in programming, as the treatment with leptin in early life affected the establishment or maintenance of DNA methylation patterns and subsequent gene expression later in life, after an environmental challenge such as obesity (Palou et al., 2018). Furthermore, leptin was shown to drive the obesity-dependent changes in global DNA methylation and gene expression in the adipose tissue of diet-induced or genetically obese mice, with evidence for obesity related global DNA hypomethylation and subsequent increased gene expression (Sonne et al., 2017). Also, the leptin-deficient ob/ob mouse was shown to have increased expression profiles of DNA methyltransferases (DNMT) 3a and 3b in adipose tissue, suggesting the potential role of leptin in the regulation of these *de novo* DNA methylation enzymes (Kamei et al., 2010; You et al., 2017). Finally, leptin has been also associated with the regulation of various miRNAs, which in turn may regulate gene expression (Derghal et al., 2015; Nakanishi et al., 2009; Sangiao-Alvarellos et al., 2014). Therefore, there is increasing evidence in the literature that supports the direct involvement of leptin in DNA methylation regulation at various cellular levels, with potential consequences for gene expression regulation.

Given the evidence here presented, it is expectable that fluctuations in ovarian leptin may affect the oocyte epigenome, through the modulation of its epigenetic machinery. Such effects on DNA *de novo* methylation could impact not only the oocyte epigenome, quality, and competence but also the embryo and its development. As a matter of fact, the epigenetic program of the germ cells, seems to be affected by leptin, with reports showing a decrease in sperm quality as a result of elevated HDAC1 and HDAC2 expression following leptin administration in rats (Almabhouh et al., 2017). Similarly, leptin treatment was shown to prevent oocyte apoptosis in buffalos against an inhibitor of the first and second-class HDACs called trichostatin A (Reza Shafiei Sheykhani et al., 2016). Hence, under conditions of dysregulated leptin signalling (hyperleptinemia or resistance) during the course of obesity, leptin may well negatively impact the establishment of the epigenetic programme, affecting oocyte development and growth. The need for more studies are therefore justified specifically on the roles of leptin on epigenetic regulations, with particular focus on any inter- or trans-generational consequences that an altered epigenome of germ cell may have, on not only the developing offspring, but also potentially subsequent generations.

## The putative role of leptin in developmental programming

It is widely accepted that obesity not only affects maternal health and reproductive outcomes but also exerts deleterious effects on the normal growth and development of the foetus (Guelinckx et al., 2008). Maternal obesity was previously associated with foetal macrosomia (Guelinckx et al., 2008), congenital anomalies (Moore et al., 2000), stillbirth, and perinatal death (Kristensen et al., 2005). On the other hand, postnatal studies revealed the association between maternal obesity, metabolic syndrome, and childhood obesity in the offspring (Stocker and Cawthorne, 2008). The nutritional and health status of the mother has always been known to be critical for foetal growth. However, the precise molecular mechanisms relating offspring predisposition to obesity and associated comorbidities to maternal obesity are still unclear. It is unclear what is the exact contribution of a low oocyte quality, or that of an altered intrauterine environment, to such effects in the offspring. Pregnancy is known to be associated with dramatic developmental plasticity, which through intrauterine adaptations determine the impact of prenatal environment and maternal metabolic performance (Santangeli et al., 2015) on the future health of the offspring (Dunkerton et al., 2022). Nonetheless, several lines of evidence support the contribution of altered gametes, rather than the intrauterine environment, to offspring predisposition to disease. For instance, studies showed that one-cell zygote, and blastocysts, from mice with diabetes retain the ability to result in congenital malformations and growth retardation in the offspring, despite being transferred into healthy pseudo-pregnant female recipients (Wyman et al., 2008). Another similar study employing embryo transfer experiments from mice fed a high-fat diet also claimed that defects observed on the foetus arose prior to the blastocyst stage and were not determined by potential changes in the uterine environment of obese mothers (Luzzo et al., 2012). However, the key question about the maternal factors carried on by the oocyte leading to altered developmental programming in the offspring remains unanswered. We speculate that conditions like hyperleptinemia or leptin resistance in obese mothers may drive such changes in the gamete with potential repercussions to the offspring.

Considering the evidence presented throughout this review, supporting the potential roles of leptin in the regulation of oocyte metabolism, mitochondrial function, and epigenetic landscape, it cannot be dismissed that leptin may play a role in determining short- and long-term health outcomes in the offspring. Leptin itself is an important contributor to metabolic disease, as increased leptin and reduced adiponectin levels have been described as a major feature of obesity that contributes to the establishment and maintenance of metabolic syndrome (Frühbeck et al., 2019). Also, environmental cues in early life, especially that of the maternal health and diet likely the state of altered leptin levels, were shown to alter epigenetic regulation in the offspring (Park et al., 2008; Pinney et al., 2011; Pinney and Simmons, 2010; Thompson and Einstein, 2010). Interestingly, studies also supported the notion of such epigenetic control through inheritance via both male and female gametes (Chen et al., 2016; Daxinger and Whitelaw, 2012; Z. J. Ge et al., 2014; Huypens et al., 2016; Rando and Simmons, 2015). Thus, changes in the epigenome and other content of the gametes, can be passed on and affect the health state of the offspring. For instance, a study involving the fertilization of gametes from mice subjected to different dietary treatment, suggested that epigenetic changes in the oocyte and sperm play an important role in the intergenerational transmission of susceptibility to obesity in the offspring (Huypens et al., 2016). Similarly, Chen and coworkers revealed more recently that reduced levels of TET3 dioxygenase in the oocytes from hyperglycemic mothers, could lead to maternally inherited glucose intolerance in the offspring in mice (X. Wu et al., 2022). This was mediated through the potential effect on the zygotic genome reprogramming via TET3-dependent DNA demethylation of genes involved in insulin secretion, sensitizing the offspring to glucose intolerance (X. Wu et al., 2022). Another study seeking to understand the oocyte-mediated effects of maternal obesity on embryo development reported that reduced levels of the Stella protein in oocytes obtained from mice fed high-fat diet, drove the genome-wide changes in methylation in the zygote, culminating in compromised adult metabolic phenotypes (Leong, 2018). Hence, specifically for the oocyte, disrupted metabolism in response to changes in metabolite availability and mitochondrial function, as the result of compromised local leptin signalling, can lead to alterations in the epigenome that may exert a detrimental effect on the developmental programming of the offspring.

In light of the intricate set of interactions between epigenetic mechanisms, metabolite availability, and gene expression regulation during embryo development, as well as the established role of leptin mediating such processes, one may anticipate that substantial evidence from studies will be generated in the soon future examining the impact of altered leptin levels on the female gamete and possible consequences for offspring health. Of important note, leptin has been already started to be recognized as a factor capable of affecting developmental programming in other contexts than obesity (Vickers et al., 2005). In fact, an optimal level of leptin in the umbilical cord blood was shown to be key for adequate intrauterine development of the foetus (Sivan et al., 1997), whereas increased leptin levels in maternal obesity were suggested to alter metabolic programming (Karakosta et al., 2013). For instance, leptin has been shown to be necessary for successful trophoblast invasion, having a mitogenic and anti-apoptotic effect in cultured human trophoblast cells (Magariños et al., 2007). Leptin levels have also been inversely correlated with placental weight (Buchbinder et al., 2001). Metabolically, leptin was shown to upregulate placental lipolysis (White et al., 2006) and stimulate the activity of amino acid transporter system A in the placental villi (Jansson et al., 2003), ensuring the adequate transfer of free fatty acids and neutral amino acids to the growing foetus. Leptin has also been suggested to control the intrauterine foetal glucogenic capacity, particularly by inhibition of endogenous glucose production towards term (Forhead et al., 2008). More generally, leptin is considered an important modulator of foetal growth and develpment (Hassink et al., 1997), controlling the proliferation of pancreatic islet cells(Islam et al., 2000), the development and migration of neuronal cells in the cerebral cortex (Udagawa et al., 2007), and the development of foetal adipose tissue and foetal length and body weight (Javaid et al., 2005; Valūnienė et al., 2007; Varvarigou et al., 1999). In fact, the synthesis and circulating levels of leptin in utero is known to be sensitive to changes in nutrients, hormones, or genetic influence (Forhead and Fowden, 2009). Hence, maternal overnutrition was reported to increase gene expression in foetal adipose tissues (Mühlhäusler et al., 2002). As a result, leptin is widely regarded as one of the main hormones capable of modulating the intrauterine environment, controlling foetal growth and development (Forhead and Fowden, 2009). Concerning the putative role of leptin in progmramming, it was shown that leptin can affect the formation and activity of hypothalamic networks in the foetus, which will dictate the regulation of appetite and energy balance in adult life (Bouret and Simerly, 2006; McMillen et al., 2005). Thus, exposure of the foetus to altered leptin levels at any critical period of development may, therefore, have important programming consequences. Additionally, new functions of leptin in milk (Palou et al., 2018) and amniotic fluid (Yau-Qiu et al., 2020) regardig early metabolic programming and metabolic health have also been reported in recent studies, in which animals showed long-term beneficial effects of leptin treatment against metabolic disease when leptin was administered during the lactation period. This portrays leptin as an important factor capable of modulating programming events at various developmental stages in the perinatal period.

When reproducing, women transfer through the gametes a complex cargo that comprises not only the genetic code but also the epigenome, proteins, metabolites, and other components relevant for embryo growth and, most importantly, capable of affecting developmental programming. The putative role of leptin in regulating the oocyte metabolome and epigenome renders this adipokine an important factor controlling the legacy of the gamete capable of affecting embryo development. Furthermore, the direct actions of leptin on the intrauterine environment and placentation also account for its putative impact on developmental programming. Finally, programming events can be also established post-natally (McMillen et al., 2005)during lactation, an equally relevant developmental timepoint concerning the hyperleptinemic environment seen during maternal obesity. It however remains, uncharacterised whether reversing leptin signalling in these developmental stages eventually rescues the phenotypic consequences in the offspring.

## Conclusion

The obesity epidemic is a global health problem with a profound impact on maternal-foetal health. Maternal obesity not only produces the usual grave outcomes of obesity but also poses significant risks to the development of the offspring both in the short and long run. This is due to the fact that the female gamete develops and matures in a physiologically altered conditions which may have impair the oocyte quality and alters its epigenome and metabolome. Given that the oocyte epigenome has the potential to control initial reprogramming events in the early embryo as well as longer-term metabolic outcomes, the oocyte legacy has the potential ability to affect predisposition to health and disease in the offspring. However, a better understanding of the maternal factors contributing to the alterations in developmental programming in the offspring is of extreme relevance. With growing evidence on the support of leptin in maintaining an optimal metabolic state, and mitochondrial function as well as a normal epigenetic landscape in the oocyte and other cellular contexts, its role as a major modulator of oocyte quality and successful embryo development seems secure. Generally, it is attractive to propose that perturbed leptin signalling observed during obesity, has detrimental effects as early as the oocyte stage, which further predisposes them to embryo developmental abnormalities and even metabolic diseases in the offspring.

## Glossary

**Table:**

**Acronym**	**Full form**
ATF4	Activating transcription factor 4
ATP	Adenosine triphosphate
BDNF	Brain-derived neurotrophic factor
BMI	Body mass index
c- JNKs	C-Jun N-terminal kinases
CCs	Cumulus cells
COCs	Cumulus-oocyte-complexes
DAG	Diacylglycerol
Db	Diabetic mouse
DIO	Diet induced obesity
DNMT	DNA methyltransferases
ERK	Extracellular signal regulated kinase
GCs	Granulosa cells
GLUT1	Glucose transporter 1
GTP	Guanosine triphosphate
H3K27Ac	Acetylation of Histone3 Lysine 27
H3K27M3	Trimethylation of Histone 3 lysine 27
H3K4M3	Trimethylation of Histone 3 lysine4
HATs	Histone acetyltransferases
HBP	Hexosamine biosynthesis pathway
HDAC5	Histone deacetylase 5
HDACs	Histone deacetylases
hESCs	Human embryonic stem cells
HFD	High fat diet
HPG-axis	Hypothalamic-pituitary-gonadal axis
IGF-1	Insulin-like growth factor 1
IKKε	I-kappa-B kinase epsilon
IP_3_	Triphosphoinositol
IRS	Insulin receptor substrate
JAK/STAT	Janus kinase /signal transducer and activator of transcription
JAK2	Janus Kinase-2
MAPK	Mitogen-activated protein kinase
MeCP2	Methyl-cpg-binding protein 2
MFN2	Mitofusin 2
miRNA	Microrna
mtDNA	Mitochondrial DNA
MyD88	Myeloid differentiation factor 88
NAD^+^	Nicotinamide adenine dinucleotide
Ob	Obese mouse
ObRb	Leptin receptor b
PCOS	Polycystic ovarian syndrome
PI3K	Phosphatidylinositol 3 kinase
PKC	Protein kinase C
PPP	Pentose phosphate pathway
PTP1B	Phosphotyrosine phosphatase-1B
PTPs	Protein tyrosine phosphatases
PUFA	Polyunsaturated fatty acids
ROS	Reactive oxygen species
SAM	S-adenosyl methionine
SGOC	Serine-glycine-one-carbon
SIRT1	Sirtuin 1
SOCS-3	Suppressor of cytokine signaling 3
SREBP-1c	Sterol regulatory element binding protein 1
STAT3	Signal transducer and activator of transcription 3
TCA	Tricarboxyllic
TET	Ten-eleven translocation
Trpm7	Transient receptor potential melastatin 7
α-KG	Alpha ketoglutarate

## References

[B001] Al Darwich A, Perreau C, Petit MH, Papillier P, Dupont J, Guillaume D, Mermillod P, Guignot F (2010). Effect of PUFA on embryo cryoresistance, gene expression and AMPKalpha phosphorylation in IVF-derived bovine embryos. Prostaglandins Other Lipid Mediat.

[B002] Allen JF, Paula WBM (2013). Mitochondrial genome function and maternal inheritance. Biochem Soc Trans.

[B003] Almabhouh F, Osman K, Ibrahim S, Gupalo S, Gnanou J, Ibrahim E, Singh HJ (2017). Melatonin ameliorates the adverse effects of leptin on sperm. Asian J Androl.

[B004] Baltz JM, Tartia AP (2010). Cell volume regulation in oocytes and early embryos: connecting physiology to successful culture media. Hum Reprod Update.

[B005] Banks AS, Davis SM, Bates SH, Myers MG (2000). Activation of downstream signals by the long form of the leptin receptor. J Biol Chem.

[B006] Bellver J, Melo MAB, Bosch E, Serra V, Remohí J, Pellicer A (2007). Obesity and poor reproductive outcome: the potential role of the endometrium. Fertil Steril.

[B007] Bence KK, Delibegovic M, Xue B, Gorgun CZ, Hotamisligil GS, Neel BG, Kahn BB (2006). Neuronal PTP1B regulates body weight, adiposity and leptin action. Nat Med.

[B008] Benkhalifa M, Ferreira YJ, Chahine H, Louanjli N, Miron P, Merviel P, Copin H (2014). Mitochondria: participation to infertility as source of energy and cause of senescence. Int J Biochem Cell Biol.

[B009] Bjørbæk C, Buchholz RM, Davis SM, Bates SH, Pierroz DD, Gu H, Neel BG, Myers MG, Flier JS (2001). Divergent roles of SHP-2 in ERK activation by leptin receptors. J Biol Chem.

[B010] Bjørbaek C, Kahn BB (2004). Leptin signaling in the central nervous system and the periphery. Recent Prog Horm Res.

[B011] Bjørbæk C, Lavery HJ, Bates SH, Olson RK, Davis SM, Flier JS, Myers MG (2000). SOCS3 mediates feedback inhibition of the leptin receptor via Tyr985. J Biol Chem.

[B012] Bjørbæk C, Uotani S, Silva B, Flier JS (1997). Divergent signaling capacities of the long and short isoforms of the leptin receptor. J Biol Chem.

[B013] Blanquer-Rossellõ MM, Santandreu FM, Oliver J, Roca P, Valle A (2015). Leptin modulates mitochondrial function, dynamics and biogenesis in MCF-7 cells. J Cell Biochem.

[B014] Bouret SG, Simerly RB (2006). Developmental programming of hypothalamic feeding circuits. Clin Genet.

[B015] Brabant G, Müller G, Horn R, Anderwald C, Roden M, Nave H (2005). Hepatic leptin signaling in obesity. FASEB J.

[B016] Brannian JD, Hansen KA (2002). Leptin and ovarian folliculogenesis: implications for ovulation induction and ART outcomes. Semin Reprod Med.

[B017] Buchbinder A, Lang U, Baker RS, Khoury JC, Mershon J, Clark KE (2001). Leptin in the ovine fetus correlates with fetal and placental size. Am J Obstet Gynecol.

[B018] Calbet JAL, MacLean DA (2002). Plasma glucagon and insulin responses depend on the rate of appearance of amino acids after ingestion of different protein solutions in humans. J Nutr.

[B019] Cardozo ER, Karmon AE, Gold J, Petrozza JC, Styer AK (2016). Reproductive outcomes in oocyte donation cycles are associated with donor BMI. Hum Reprod.

[B020] Castracane V, Henson MC (2003). Leptin and reproduction..

[B021] Cervero A, Horcajadas JA, Martín J, Pellicer A, Simón C (2004). The leptin system during human endometrial receptivity and preimplantation development. J Clin Endocrinol Metab.

[B022] Chen Q, Yan W, Duan E (2016). Epigenetic inheritance of acquired traits through sperm RNAs and sperm RNA modifications. Nat Rev Genet.

[B023] Cheng A, Uetani N, Simoncic PD, Chaubey VP, Lee-Loy A, McGlade CJ, Kennedy BP, Tremblay ML (2002). Attenuation of leptin action and regulation of obesity by protein tyrosine phosphatase 1B. Dev Cell.

[B024] Childs G, Odle AK, MacNicol MC, MacNicol AM (2021). The importance of leptin to reproduction. Endocrinology.

[B025] Chou SH, Mantzoros C (2014). 20 years of leptin: role of leptin in human reproductive disorders. J Endocrinol.

[B026] Considine R, Sinha MK, Heiman ML, Kriauciunas A, Stephens TW, Nyce MR, Ohannesian JP, Marco CC, McKee LJ, Bauer TL, Caro JF (1996). Serum immunoreactive-leptin concentrations in normal-weight and obese humans. N Engl J Med.

[B027] Cui Y, Miyoshi K, Claudio E, Siebenlist UK, Gonzalez FJ, Flaws J, Wagner KU, Hennighausen L (2002). Loss of the peroxisome proliferation-activated receptor gamma (PPARgamma) does not affect mammary development and propensity for tumor formation but leads to reduced fertility. J Biol Chem.

[B028] D’souza AM, Neumann UH, Glavas MM, Kieffer TJ (2017). The glucoregulatory actions of leptin. Mol Metab.

[B029] Dağ ZÖ, Dilbaz B (2015). Impact of obesity on infertility in women. J Turk Ger Gynecol Assoc.

[B030] Dalamaga M, Chou SH, Shields K, Papageorgiou P, Polyzos SA, Mantzoros CS (2013). Leptin at the intersection of neuroendocrinology and metabolism: current evidence and therapeutic perspectives. Cell Metab.

[B031] Daxinger L, Whitelaw E (2012). Understanding transgenerational epigenetic inheritance via the gametes in mammals. Nat Rev Genet.

[B032] Derghal A, Djelloul M, Airault C, Pierre C, Dallaporta M, Troadec JD, Tillement V, Tardivel C, Bariohay B, Trouslard J, Mounien L (2015). Leptin is required for hypothalamic regulation of miRNA stargeting POMC 3′UTR. Front Cell Neurosci.

[B033] Downs SM, Hudson ED (2000). Energy substrates and the completion of spontaneous meiotic maturation. Zygote.

[B034] Downs SM, Mosey JL, Klinger J (2009). Fatty acid oxidation and meiotic resumption in mouse oocytes. Mol Reprod Dev.

[B035] Dunkerton S, Mrcog M, Aiken C, Bchir M, Mrcp M (2022). Impact of the intrauterine environment on future reproductive and metabolic health. Obstet Gynaecol.

[B036] Dunning KR, Akison LK, Russell DL, Norman RJ, Robker RL (2011). Increased beta-oxidation and improved oocyte developmental competence in response to L-Carnitine during ovarian in vitro follicle development in mice. Biol Reprod.

[B037] Dunning KR, Russell DL, Robker RL (2014). Lipids and oocyte developmental competence: the role of fatty acids and β-oxidation. Reproduction.

[B038] Enriori PJ, Evans AE, Sinnayah P, Cowley MA (2006). Leptin resistance and obesity. Obesity.

[B039] Enriori PJ, Evans AE, Sinnayah P, Jobst EE, Tonelli-Lemos L, Billes SK, Glavas MM, Grayson BE, Perello M, Nillni EA, Grove KL, Cowley MA (2007). Diet-induced obesity causes severe but reversible leptin resistance in arcuate melanocortin neurons. Cell Metab.

[B040] Eppig JJ, Marin-Bivens C, Viveiros MM, de la Fuente R, Leung PCK, Adashi EY (2004). The ovary..

[B041] Farooqi IS, O’Rahilly S (2009). Leptin: a pivotal regulator of human energy homeostasis. Am J Clin Nutr.

[B042] Ferguson EM, Leese HJ (2006). A potential role for triglyceride as an energy source during bovine oocyte maturation and early embryo development. Mol Reprod Dev.

[B043] Fiedor E, Zajda K, Gregoraszczuk EL (2018). Leptin receptor antagonists’ action on HDAC expression eliminating the negative effects of leptin in ovarian cancer. Cancer Genomics Proteomics.

[B044] Flaus A, Owen-Hughes T (2011). Mechanisms for ATP-dependent chromatin remodelling: the means to the end. FEBS J.

[B045] Forhead AJ, Fowden AL (2009). The hungry fetus? Role of leptin as a nutritional signal before birth. J Physiol.

[B046] Forhead AJ, Lamb CA, Franko KL, O’Connor DM, Wooding FBP, Cripps RL, Ozanne S, Blache D, Shen QW, Du M, Fowden AL (2008). Role of leptin in the regulation of growth and carbohydrate metabolism in the ovine fetus during late gestation. J Physiol.

[B047] Friedman JM, Halaas JL (1998). Leptin and the regulation of body weight in mammals. Nature.

[B048] Frühbeck G, Catalán V, Rodríguez A, Ramírez B, Becerril S, Salvador J, Colina I, Gómez-Ambrosi J (2019). Adiponectin-leptin ratio is a functional biomarker of adipose tissue inflammation. Nutrients.

[B049] Galvão A, Henriques S, Pestka D, Lukasik K, Skarzynski D, Mateus LM, Ferreira-Dias GML (2012). Equine luteal function regulation may depend on the interaction between cytokines and vascular endothelial growth factor: an in vitro study. Biol Reprod.

[B050] Ge H, Tollner TL, Hu Z, Dai M, Li X, Guan H, Shan D, Zhang X, Lv J, Huang C, Dong Q (2012). The importance of mitochondrial metabolic activity and mitochondrial DNA replication during oocyte maturation in vitro on oocyte quality and subsequent embryo developmental competence. Mol Reprod Dev.

[B051] Ge ZJ, Luo SM, Lin F, Liang QX, Huang L, Wei YC, Hou Y, Han ZM, Schatten H, Sun QY (2014). DNA methylation in oocytes and liver of female mice and their offspring: effects of high-fat-diet-induced obesity. Environ Health Perspect.

[B052] Ge ZJ, Schatten H, Zhang CL, Sun QY (2015). Oocyte ageing and epigenetics. Reproduction.

[B053] Gilbody S, Lewis S, Lightfoot T (2007). Methylenetetrahydrofolate reductase (MTHFR) genetic polymorphisms and psychiatric disorders: a HuGE review. Am J Epidemiol.

[B054] Grewal S, Gubbi S, Fosam A, Sedmak C, Sikder S, Talluru H, Brown RJ, Muniyappa R (2020). Metabolomic analysis of the effects of leptin replacement therapy in patients with lipodystrophy. J Endocr Soc.

[B055] Gu L, Liu H, Gu X, Boots C, Moley KH, Wang Q (2015). Metabolic control of oocyte development: linking maternal nutrition and reproductive outcomes. Cell Mol Life Sci.

[B056] Guelinckx I, Devlieger R, Beckers K, Vansant G (2008). Maternal obesity: pregnancy complications, gestational weight gain and nutrition. Obes Rev.

[B057] Harris SE, Adriaens I, Leese HJ, Gosden RG, Picton HM (2007). Carbohydrate metabolism by murine ovarian follicles and oocytes grown in vitro. Reproduction.

[B058] Hassink SG, de Lancey E, Sheslow D, Smith-Kirwin SM, O’Connor DM, Considine R, Opentanova I, Dostal K, Spear ML, Leef K, Ash M, Spitzer AR, Funanage VL (1997). Placental leptin: an important new growth factor in intrauterine and neonatal development?. Pediatrics.

[B059] Hausman GJ, Barb CR, Lents CA (2012). Leptin and reproductive function. Biochimie.

[B060] He M, Zhang T, Yang Y, Wang C (2021). Mechanisms of oocyte maturation and related epigenetic regulation. Front Cell Dev Biol.

[B061] Hegyi K, Fülöp K, Kovács K, Tóth S, Falus A (2004). Leptin-induced signal transduction pathways. Cell Biol Int.

[B062] Henry BA, Andrews ZB, Rao A, Clarke IJ (2011). Central leptin activates mitochondrial function and increases heat production in skeletal muscle. Endocrinology.

[B063] Hong J, Lee E (2007). Intrafollicular amino acid concentration and the effect of amino acids in a defined maturation medium on porcine oocyte maturation, fertilization, and preimplantation development. Theriogenology.

[B064] Hruby A, Hu FB (2015). The Epidemiology of Obesity: A Big Picture. PharmacoEconomics.

[B065] Hunt PA, Hassold TJ (2008). Human female meiosis: what makes a good egg go bad?. Trends Genet.

[B066] Huypens P, Sass S, Wu M, Dyckhoff D, Tschöp M, Theis F, Marschall S, de Angelis MH, Beckers J (2016). Epigenetic germline inheritance of diet-induced obesity and insulin resistance. Nat Genet.

[B067] Igosheva N, Abramov AY, Poston L, Eckert JJ, Fleming TP, Duchen MR, McConnell J (2010). Maternal diet-induced obesity alters mitochondrial activity and redox status in mouse oocytes and zygotes. PLoS One.

[B068] Islam MS, Sjöholm Å, Emilsson V (2000). Fetal pancreatic islets express functional leptin receptors and leptin stimulates proliferation of fetal islet cells. Int J Obes.

[B069] Jansson N, Greenwood SL, Johansson BR, Powell TL, Jansson T (2003). Leptin stimulates the activity of the system A amino acid transporter in human placental villous fragments. J Clin Endocrinol Metab.

[B070] Javaid MK, Godfrey KM, Taylor P, Robinson SM, Crozier SR, Dennison EM, Robinson JS, Breier BR, Arden NK, Cooper C (2005). Umbilical cord leptin predicts neonatal bone mass. Calcif Tissue Int.

[B071] Johnson MT, Freeman EA, Gardner DK, Hunt PA (2007). Oxidative metabolism of pyruvate is required for meiotic maturation of murine oocytes in vivo. Biol Reprod.

[B072] Kalive M, Faust JJ, Koeneman BA, Capco DG (2010). Involvement of the PKC family in regulation of early development. Mol Reprod Dev.

[B073] Kamei Y, Suganami T, Ehara T, Kanai S, Hayashi K, Yamamoto Y, Miura S, Ezaki O, Okano M, Ogawa Y (2010). Increased expression of DNA methyltransferase 3a in obese adipose tissue: studies with transgenic mice. Obesity.

[B074] Karakosta P, Georgiou V, Fthenou E, Papadopoulou E, Roumeliotaki T, Margioris A, Castanas E, Kampa M, Kogevinas M, Chatzi L (2013). Maternal weight status, cord blood leptin and fetal growth: a prospective mother-child cohort study (Rhea study). Paediatr Perinat Epidemiol.

[B075] Khan R, Jiang X, Hameed U, Shi Q (2021). Role of lipid metabolism and signaling in mammalian oocyte maturation, quality, and acquisition of competence. Front Cell Dev Biol.

[B076] Kirchberg FF, Brandt S, Moß A, Peissner W, Koenig W, Rothenbacher D, Brenner H, Koletzko B, Hellmuth C, Wabitsch M (2017). Metabolomics reveals an entanglement of fasting leptin concentrations with fatty acid oxidation and gluconeogenesis in healthy children. PLoS One.

[B077] Kirillova A, Smitz JEJ, Sukhikh GT, Mazunin I (2021). The role of mitochondria in oocyte maturation. Cells.

[B078] Koch C, Augustine RA, Steger J, Ganjam GK, Benzler J, Pracht C, Lowe C, Schwartz MW, Shepherd PR, Anderson GM, Grattan DR, Tups A (2010). Leptin rapidly improves glucose homeostasis in obese mice by increasing hypothalamic insulin sensitivity. J Neurosci.

[B079] Kooistra SM, Helin K (2012). Molecular mechanisms and potential functions of histone demethylases. Nat Rev Mol Cell Biol.

[B080] Kristensen J, Vestergaard M, Wisborg K, Kesmodel U, Secher NJ (2005). Pre-pregnancy weight and the risk of stillbirth and neonatal death. BJOG.

[B081] Kurus M, Karakaya C, Karalok MH, To G, Johnson J (2013). The control of oocyte survival by intrinsic and extrinsic factors. Adv Exp Med Biol.

[B082] Kyrou I, Randeva HS, Tsigos C, Kaltsas G, Weickert MO, Feingold KR, Anawalt B, Blackman MR, Boyce A, Chrousos G, Corpas E, Herder WW, Dhatariya K, Hofland J, Dungan K, Hofland J, Kalra S, Kaltsas G, Kapoor N, Koch C, Kopp P, Korbonits M, Kovacs CS, Kuohung W, Laferrère B, Levy M, McGee EA, McLachlan R, New M, Purnell J, Sahay R, Singer F, Sperling MA, Stratakis CA, Trence DL, Wilson DP (2018). Endotext.

[B083] Lawler K, Huang-Doran I, Sonoyama T, Collet TH, Keogh JM, Henning E, O’Rahilly S, Bottolo L, Farooqi IS (2020). Leptin-mediated changes in the human metabolome. J Clin Endocrinol Metab.

[B084] Leong I (2018). Link between maternal obesity and offspring is STELLA. Nat Rev Endocrinol.

[B085] Li L, Zhu S, Shu W, Guo Y, Guan Y, Zeng J, Wang H, Han L, Zhang J, Liu X, Li C, Hou X, Gao M, Ge J, Ren C, Zhang H, Schedl T, Guo X, Chen M, Wang Q (2020). Characterization of metabolic patterns in mouse oocytes during meiotic maturation. Mol Cell.

[B086] Lin J, Barb CR, Matteri RL, Kraeling RR, Chen X, Meinersmann RJ, Rampacek GB (2000). Long form leptin receptor mRNA expression in the brain, pituitary, and other tissues in the pig. Domest Anim Endocrinol.

[B087] Liu J, Yang X, Yu S, Zheng R (2018). The leptin resistance. Adv Exp Med Biol.

[B088] Liu T, Qu J, Tian M, Yang R, Song X, Li R, Yan J, Qiao J (2022). Lipid metabolic process involved in oocyte maturation during folliculogenesis. Front Cell Dev Biol.

[B089] Luo GF, Yu TY, Wen XH, Li Y, Yang GS (2008). Alteration of mitochondrial oxidative capacity during porcine preadipocyte differentiation and in response to leptin. Mol Cell Biochem.

[B090] Luzzo KM, Wang Q, Purcell SH, Chi M, Jimenez PT, Grindler N, Schedl T, Moley KH (2012). High fat diet induced developmental defects in the mouse: oocyte meiotic aneuploidy and fetal growth retardation/brain defects. PLoS One.

[B091] Maffei M, Halaas J, Ravussin E, Pratley RE, Lee GH, Zhang Y, Fei H, Kim S, Lallone R, Ranganathan S, Kern PA, Friedman JM (1995). Leptin levels in human and rodent: measurement of plasma leptin and ob RNA in obese and weight-reduced subjects. Nat Med.

[B092] Magariños MP, Sánchez-Margalet V, Kotler M, Calvo JC, Varone CL (2007). Leptin promotes cell proliferation and survival of trophoblastic cells. Biol Reprod.

[B093] Mantzoros CS, Cramer DW, Liberman RF, Barbieri RL (2000). Predictive value of serum and follicular fluid leptin concentrations during assisted reproductive cycles in normal women and in women with the polycystic ovarian syndrome. Hum Reprod.

[B094] Mark AL, Correia MLG, Rahmouni K, Haynes WG (2002). Selective leptin resistance: a new concept in leptin physiology with cardiovascular implications. J Hypertens.

[B095] Matarese G, Procaccini C, De Rosa V, Castracane VD, Henson MC (2006). Leptin..

[B096] Matilainen O, Quirós PM, Auwerx J (2017). Mitochondria and epigenetics: crosstalk in homeostasis and stress. Trends Cell Biol.

[B097] McKeegan PJ, Sturmey RG (2011). The role of fatty acids in oocyte and early embryo development. Reprod Fertil Dev.

[B098] McMillen IC, Adam CL, Mühlhäusler BS (2005). Early origins of obesity: programming the appetite regulatory system. J Physiol.

[B099] McPherson NO, Bell VG, Zander-Fox DL, Fullston T, Wu LL, Robker RL, Lane M (2015). When two obese parents are worse than one! Impacts on embryo and fetal development. Am J Physiol Endocrinol Metab.

[B100] Menzies KJ, Zhang H, Katsyuba E, Auwerx J (2016). Protein acetylation in metabolism-metabolites and cofactors. Nat Rev Endocrinol.

[B101] Miller KK, Parulekar MS, Schoenfeld E, Anderson E, Hubbard J, Klibanski A, Grinspoon SK (1998). Decreased leptin levels in normal weight women with hypothalamic amenorrhea: the effects of body composition and nutritional intake. J Clin Endocrinol Metab.

[B102] Minokoshi Y, Kim YB, Peroni OD, Fryer LGD, Müller C, Carling D, Kahn BB (2002). Leptin stimulates fatty-acid oxidation by activating AMP-activated protein kinase. Nature.

[B103] Minokoshi Y, Toda C, Okamoto S (2012). Regulatory role of leptin in glucose and lipid metabolism in skeletal muscle. Indian J Endocrinol Metab.

[B104] Moley KH, Chi MMY, Knudson CM, Korsmeyer SJ, Mueckler MM (1998). Hyperglycemia induces apoptosis in pre-implantation embryos through cell death effector pathways. Nat Med.

[B105] Montgomery DC, Sorum AW, Guasch L, Nicklaus MC, Meier JL (2015). Metabolic regulation of histone acetyltransferases by endogenous Acyl-CoA cofactors. Chem Biol.

[B106] Moore LL, Singer MR, Bradlee ML, Rothman KJ, Milunsky A (2000). A prospective study of the risk of congenital defects associated with maternal obesity and diabetes mellitus. Epidemiology.

[B107] Morgan DA, Thedens DR, Weiss R, Rahmouni K (2008). Mechanisms mediating renal sympathetic activation to leptin in obesity. Am J Physiol Regul Integr Comp Physiol.

[B108] Morrish F, Noonan J, Perez-Olsen C, Gafken PR, Fitzgibbon M, Kelleher J, VanGilst M, Hockenbery D (2010). Myc-dependent mitochondrial generation of acetyl-CoA contributes to fatty acid biosynthesis and histone acetylation during cell cycle entry. J Biol Chem.

[B109] Moslehi N, Shab-Bidar S, Tehrani FR, Mirmiran P, Azizi F (2018). Is ovarian reserve associated with body mass index and obesity in reproductive aged women? A meta-analysis. Menopause.

[B110] Mühlhäusler BS, Roberts CT, McFarlane JR, Kauter KG, McMillen IC (2002). Fetal leptin is a signal of fat mass independent of maternal nutrition in ewes fed at or above maintenance energy requirements. Biol Reprod.

[B111] Münzberg H, Flier JS, Bjørbæk C (2004). Region-specific leptin resistance within the hypothalamus of diet-induced obese mice. Endocrinology.

[B112] Myers MG, Leibel RL, Seeley RJ, Schwartz MW (2010). Obesity and leptin resistance: distinguishing cause from effect. trends endocrinol metab.

[B113] Myers MG (2004). Leptin receptor signaling and the regulation of mammalian physiology. Recent Prog Horm Res.

[B114] Myers MP, Andersen JN, Cheng A, Tremblay ML, Horvath CM, Parisien JP, Salmeen A, Barford D, Tonks NK (2001). TYK2 and JAK2 are substrates of protein-tyrosine phosphatase 1B. J Biol Chem.

[B115] Nakanishi N, Nakagawa Y, Tokushige N, Aoki N, Matsuzaka T, Ishii K, Yahagi N, Kobayashi K, Yatoh S, Takahashi A, Suzuki H, Urayama O, Yamada N, Shimano H (2009). The up-regulation of microRNA-335 is associated with lipid metabolism in liver and white adipose tissue of genetically obese mice. Biochem Biophys Res Commun.

[B116] Orsi NM, Gopichandran N, Leese HJ, Picton HM, Harris SE (2005). Fluctuations in bovine ovarian follicular fluid composition throughout the oestrous cycle. Reproduction.

[B117] Ozcan L, Ergin AS, Lu A, Chung J, Sarkar S, Nie D, Myers MG, Ozcan U (2009). Endoplasmic reticulum stress plays a central role in development of leptin resistance. Cell Metab.

[B118] Palou M, Picó C, McKay JA, Sánchez J, Priego T, Mathers JC, Palou A (2011). Protective effects of leptin during the suckling period against later obesity may be associated with changes in promoter methylation of the hypothalamic pro-opiomelanocortin gene. Br J Nutr.

[B119] Palou M, Picó C, Palou A (2018). Leptin as a breast milk component for the prevention of obesity. Nutr Rev.

[B120] Park J, Kusminski CM, Chua SC, Scherer PE (2010). Leptin receptor signaling supports cancer cell metabolism through suppression of mitochondrial respiration in vivo. Am J Pathol.

[B121] Park JH, Stoffers DA, Nicholls RD, Simmons RA (2008). Development of type 2 diabetes following intrauterine growth retardation in rats is associated with progressive epigenetic silencing of Pdx1. J Clin Invest.

[B122] Paula-Lopes FF, Boelhauve M, Habermann FA, Sinowatz F, Wolf E (2007). Leptin promotes meiotic progression and developmental capacity of bovine oocytes via cumulus cell-independent and-dependent mechanisms. Biol Reprod.

[B123] Pelland AMD, Corbett HE, Baltz JM (2009). Amino acid transport mechanisms in mouse oocytes during growth and meiotic maturation. Biol Reprod.

[B124] Pereira S, Cline DL, Glavas MM, Covey SD, Kieffer TJ (2021). Tissue-specific effects of leptin on glucose and lipid metabolism. Endocr Rev.

[B125] Pérez-Pérez A, Sánchez-Jiménez F, Maymó J, Dueñas JL, Varone C, Sánchez-Margalet V (2015). Role of leptin in female reproduction. Clin Chem Lab Med.

[B126] Pinney SE, Jaeckle Santos LJ, Han Y, Stoffers DA, Simmons RA (2011). Exendin-4 increases histone acetylase activity and reverses epigenetic modifications that silence Pdx1 in the intrauterine growth retarded rat. Diabetologia.

[B127] Pinney SE, Simmons RA (2010). Epigenetic mechanisms in the development of type 2 diabetes. Trends Endocrinol Metab.

[B128] Pirola L, Balcerczyk A, Okabe J, El-Osta A (2010). Epigenetic phenomena linked to diabetic complications. Nat Rev Endocrinol.

[B129] Poretsky L, Cataldo NA, Rosenwaks Z, Giudice LC (1999). The insulin-related ovarian regulatory system in health and disease. Endocr Rev.

[B130] Qi L, Chen X, Wang J, Lv B, Zhang J, Ni B, Xue Z (2019). Mitochondria: the panacea to improve oocyte quality?. Ann Transl Med.

[B131] Rabe K, Lehrke M, Parhofer KG, Broedl UC (2008). Adipokines and insulin resistance. Mol Med.

[B132] Rando OJ, Simmons RA (2015). I’m eating for two: parental dietary effects on offspring metabolism. Cell.

[B133] Reidy SP, Weber JM (2000). Leptin: an essential regulator of lipid metabolism. Comp Biochem Physiol A Mol Integr Physiol.

[B134] Reina-Campos M, Diaz-Meco MT, Moscat J (2020). The complexity of the serine glycine one-carbon pathway in cancer. J Cell Biol.

[B135] Robker RL, Akison LK, Bennett BD, Thrupp PN, Chura LR, Russell DL, Lane M, Norman RJ (2009). Obese women exhibit differences in ovarian metabolites, hormones, and gene expression compared with moderate-weight women. J Clin Endocrinol Metab.

[B136] Robker RL, Wu LLY, Yang X (2011). Inflammatory pathways linking obesity and ovarian dysfunction. J Reprod Immunol.

[B137] Rooke JA, Ainslie A, Watt RG, Alink FM, McEvoy TG, Sinclair KD, Garnsworthy PC, Webb R (2009). Dietary carbohydrates and amino acids influence oocyte quality in dairy heifers. Reprod Fertil Dev.

[B138] Ruiz-Cortés ZT, Men T, Palin MF, Downey BR, Lacroix DA, Murphy BD (2000). Porcine leptin receptor: molecular structure and expression in the ovary. Mol Reprod Dev.

[B139] Russell DL, Gilchrist RB, Brown HM, Thompson JG (2016). Bidirectional communication between cumulus cells and the oocyte: old hands and new players?. Theriogenology.

[B140] Ryan NK, Woodhouse CM, van der Hoek KH, Gilchrist RB, Armstrong DT, Norman RJ (2002). Expression of leptin and its receptor in the murine ovary: possible role in the regulation of oocyte maturation. Biol Reprod.

[B141] Sáinz N, Barrenetxe J, Moreno-Aliaga MJ, Martínez JA (2015). Leptin resistance and diet-induced obesity: central and peripheral actions of leptin. Metabolism.

[B142] Saito T, Hiroi M, Kato T (1994). Development of glucose utilization studied in single oocytes and preimplantation embryos from mice. Biol Reprod.

[B143] Sampath H, Ntambi JM (2005). Polyunsaturated fatty acid regulation of genes of lipid metabolism. Annu Rev Nutr.

[B144] Sangiao-Alvarellos S, Pena-Bello L, Manfredi-Lozano M, Tena-Sempere M, Cordido F (2014). Perturbation of hypothalamic microRNA expression patterns in male rats after metabolic distress: impact of obesity and conditions of negative energy balance. Endocrinology.

[B145] Santangeli L, Sattar N, Huda SS (2015). Impact of maternal obesity on perinatal and childhood outcomes. Best Pract Res Clin Obstet Gynaecol.

[B146] Schatten H, Sun Q-Y, Prather R (2014). The impact of mitochondrial function/dysfunction on IVF and new treatment possibilities for infertility. Reprod Biol Endocrinol.

[B147] Shafiei Sheykhani HR, Batavani RA, Najafi GR (2016). Protective effect of leptin on induced apoptosis with trichostatin A on buffalo oocytes. Vet Res Forum.

[B148] Shen L, Cordero JF, Wang JS, Shen Y, Li S, Liang L, Zou Z, Li C (2019). Association between genetically determined leptin and blood lipids considering alcohol consumption: a Mendelian randomisation study. BMJ Open.

[B149] Short KR, Chadwick JQ, Teague AM, Tullier MA, Wolbert L, Coleman C, Copeland KC (2019). Effect of obesity and exercise training on plasma amino acids and amino metabolites in american indian adolescents. J Clin Endocrinol Metab.

[B150] Silva E, Paczkowski M, Krisher RL (2012). The effect of leptin on maturing porcine oocytes is dependent on glucose concentration. Mol Reprod Dev.

[B151] Sinclair KD, Kuran M, Gebbie FE, Webb R, McEvoy TG (2000). Nitrogen metabolism and fertility in cattle: II. Development of oocytes recovered from heifers offered diets differing in their rate of nitrogen release in the rumen. J Anim Sci.

[B152] Sirard MA (2011). Follicle environment and quality of in vitro matured oocytes. J Assist Reprod Genet.

[B153] Sivan E, Lin WM, Homko CJ, Reece EA, Boden G (1997). Leptin is present in human cord blood. Diabetes.

[B154] Smith BC, Denu JM (2009). Chemical mechanisms of histone lysine and arginine modifications. Biochim Biophys Acta.

[B155] Song NY, Lee YH, Na HK, Baek JH, Surh YJ (2018). Leptin induces SIRT1 expression through activation of NF-E2-related factor 2: implications for obesity-associated colon carcinogenesis. Biochem Pharmacol.

[B156] Songsasen N, Wildt DE (2007). Oocyte biology and challenges in developing in vitro maturation systems in the domestic dog. Anim Reprod Sci.

[B157] Sonne SB, Yadav R, Yin G, Dalgaard MD, Myrmel LS, Gupta R, Wang J, Madsen L, Kajimura S, Kristiansen K (2017). Obesity is associated with depot-specific alterations in adipocyte DNA methylation and gene expression. Adipocyte.

[B158] Sperber H, Mathieu J, Wang Y, Ferreccio A, Hesson J, Xu Z, Fischer KA, Devi A, Detraux D, Gu H, Battle SL, Showalter M, Valensisi C, Bielas JH, Ericson NG, Margaretha L, Robitaille AM, Margineantu D, Fiehn O, Hockenbery D, Blau CA, Raftery D, Margolin AA, Hawkins RD, Moon RT, Ware CB, Ruohola-Baker H (2015). The metabolome regulates the epigenetic landscape during naïve to primed human embryonic stem cell transition. Nat Cell Biol.

[B159] Spinelli JB, Haigis MC (2018). The multifaceted contributions of mitochondria to cellular metabolism. Nat Cell Biol.

[B160] Stocker CJ, Cawthorne MA (2008). The influence of leptin on early life programming of obesity. Trends Biotechnol.

[B161] Strum JC, Swenson KI, Turner JE, Bell RM (1995). Ceramide triggers meiotic cell cycle progression in Xenopus oocytes. A potential mediator of progesterone-induced maturation. J Biol Chem.

[B162] Sturmey RG, Brison DR, Leese HJ (2008). Assessing embryo viability by measurement of amino acid turnover. Reprod Biomed Online.

[B163] Su Y-Q, Sugiura K, Eppig JJ (2009). mouse oocyte control of granulosa cell development and function: paracrine regulation of cumulus cell metabolism. Semin Reprod Med.

[B164] Sugiura K, Eppig JJ (2005). Control of metabolic cooperativity between oocytes and their companion granulosa cells by mouse oocytes. Reprod Fertil Dev.

[B165] Sutton-McDowall ML, Gilchrist RB, Thompson JG (2010). The pivotal role of glucose metabolism in determining oocyte developmental competence. Reproduction.

[B166] Tartaglia LA, Dembski M, Weng X, Deng N, Culpepper J, Devos R, Richards GJ, Campfield LA, Clark FT, Deeds J, Muir C, Sanker S, Moriarty A, Moore KJ, Smutko JS, Mays GG, Wool EA, Monroe CA, Tepper RI (1995). Identification and expression cloning of a leptin receptor, OB-R. Cell.

[B167] Tchio CM, Harbuzariu A, Harmon T, Beech D, Gonzalez-Perez R (2016). Abstract 1901: leptin modulation of PCSC, HDAC, and microRNA in pancreatic adenocarcinoma. Cancer Res.

[B168] Thompson RF, Einstein FH (2010). Epigenetic basis for fetal origins of age-related disease. J Womens Health (Larchmt).

[B169] Tong Q, Xu Y (2012). Central Leptin Regulation of Obesity and Fertility. Curr Obes Rep.

[B170] Tups A (2009). Physiological Models of Leptin Resistance. J Neuroendocrinol.

[B171] Udagawa J, Hatta T, Hashimoto R, Otani H (2007). Roles of leptin in prenatal and perinatal brain development. Congenit Anom (Kyoto).

[B172] Valūnienė M, Verkauskienė R, Boguszewski M, Dahlgren J, Lašienė D, Lašas L, Wikland KA (2007). Leptin levels at birth and in early postnatal life in small- and appropriate-for-gestational-age infants. Medicina.

[B173] van Winkle LJ (2001). Amino acid transport regulation and early embryo development. Biol Reprod.

[B174] Varvarigou A, Mantzoros CS, Beratis NG (1999). Cord blood leptin concentrations in relation to intrauterine growth. Clin Endocrinol.

[B175] Vickers MH, Gluckman PD, Coveny AH, Hofman PL, Cutfield WS, Gertler A, Breier BH, Harris M (2005). Neonatal leptin treatment reverses developmental programming. Endocrinology.

[B176] Wai T, Ao A, Zhang X, Cyr D, Dufort D, Shoubridge EA (2010). The role of mitochondrial DNA copy number in mammalian fertility. Biol Reprod.

[B177] Watson AJ (2007). Oocyte cytoplasmic maturation: a key mediator of oocyte and embryo developmental competence. J Anim Sci.

[B178] Welt CK, Chan JL, Bullen J, Murphy R, Smith P, DePaoli AM, Karalis A, Mantzoros CS (2004). Recombinant human leptin in women with hypothalamic amenorrhea. N Engl J Med.

[B179] Whidden L, Martel J, Rahimi S, Chaillet JR, Chan D, Trasler JM (2016). Compromised oocyte quality and assisted reproduction contribute to sex-specific effects on offspring outcomes and epigenetic patterning. Hum Mol Genet.

[B180] White V, González E, Capobianco E, Pustovrh C, Martínez N, Higa R, Baier M, Jawerbaum A (2006). Leptin modulates nitric oxide production and lipid metabolism in human placenta. Reprod Fertil Dev.

[B181] Wołodko K, Castillo‐fernandez J, Kelsey G, Galvão A (2021). Revisiting the impact of local leptin signaling in folliculogenesis and oocyte maturation in obese mothers. Int J Mol Sci.

[B182] Wołodko K, Walewska E, Adamowski M, Castillo-Fernandez J, Kelsey G, Galvão A (2020). Leptin resistance in the ovary of obese mice is associated with profound changes in the transcriptome of cumulus cells. Cell Physiol Biochem.

[B183] Wróblewski A, Strycharz J, Świderska E, Drewniak K, Drzewoski J, Szemraj J, Kasznicki J, Śliwińska A (2019). Molecular insight into the interaction between epigenetics and leptin in metabolic disorders. Nutrients.

[B184] Wu LLY, Dunning KR, Yang X, Russell DL, Lane M, Norman RJ, Robker RL (2010). High-fat diet causes lipotoxicity responses in cumulus-oocyte complexes and decreased fertilization rates. Endocrinology.

[B185] Wu X, Xu S, Weng J (2022). Hyperglycemia-mediated oocyte TET3 insufficiency predisposes offspring to glucose intolerance. J Diabetes Investig.

[B186] Wyman A, Pinto AB, Sheridan R, Moley KH (2008). One-cell zygote transfer from diabetic to nondiabetic mouse results in congenital malformations and growth retardation in offspring. Endocrinology.

[B187] Yamagishi SI, Edelstein D, Du XL, Kaneda Y, Guzmán M, Brownlee M (2001). Leptin induces mitochondrial superoxide production and monocyte chemoattractant protein-1 expression in aortic endothelial cells by increasing fatty acid oxidation via protein kinase A. J Biol Chem.

[B188] Yang X, Wu LL, Chura LR, Liang X, Lane M, Norman RJ, Robker RL (2012). Exposure to lipid-rich follicular fluid is associated with endoplasmic reticulum stress and impaired oocyte maturation in cumulus-oocyte complexes. Fertil Steril.

[B189] Yau-Qiu ZX, Picó C, Rodríguez AM, Palou A (2020). Leptin distribution in rat foetal and extraembryonic tissues in late gestation: a physiological view of amniotic fluid leptin. Nutrients.

[B190] Yeung BHY, Griffiths K, Berger L, Paudel O, Shin MK, Rui L, Sham JSK, Polotsky VY, Tang WY (2021). Leptin induces epigenetic regulation of transient receptor potential melastatin 7 in rat adrenal pheochromocytoma cells. Am J Respir Cell Mol Biol.

[B191] Yin S, Jiang X, Jiang H, Gao Q, Wang F, Fan S, Khan T, Jabeen N, Khan M, Ali A, Xu P, Pandita TK, Fan H-Y, Zhang Y, Shi Q (2017). Histone acetyltransferase KAT8 is essential for mouse oocyte development by regulating reactive oxygen species levels. Development.

[B192] You D, Nilsson E, Tenen DE, Lyubetskaya A, Lo JC, Jiang R, Deng J, Dawes BA, Vaag A, Ling C, Rosen ED, Kang S (2017). Dnmt3a is an epigenetic mediator of adipose insulin resistance. eLife.

[B193] Zhang F, Chen Y, Heiman M, DiMarchi R (2005). Leptin: structure, function and biology. Vitam Horm.

[B194] Zhang LH, Tan XY, Wu K, Zhuo MQ, Song YF, Chen QL (2015). Regulation and mechanism of leptin on lipid metabolism in ovarian follicle cells from yellow catfish Pelteobagrus fulvidraco. Gen Comp Endocrinol.

[B195] Zhang Y, Proenca R, Maffei M, Barone M, Leopold L, Friedman JM (1994). Positional cloning of the mouse obese gene and its human homologue. Nature.

[B196] Zhou Y, Qiu L, Xiao Q, Wang Y, Meng X, Xu R, Wang S, Na R (2013). Obesity and diabetes related plasma amino acid alterations. Clin Biochem.

